# Multiple regression model to analyze the total LOS for patients undergoing laparoscopic appendectomy

**DOI:** 10.1186/s12911-022-01884-9

**Published:** 2022-05-24

**Authors:** Teresa Angela Trunfio, Arianna Scala, Cristiana Giglio, Giovanni Rossi, Anna Borrelli, Maria Romano, Giovanni Improta

**Affiliations:** 1 Department of Advanced Biomedical Sciences, University Hospital of Naples ‘Federico II’, Naples, Italy; 2 Department of Public Health, University of Naples “Federico II”, Naples, Italy; 3 University of Rome “La Sapienza”, Rome, Italy; 4 “San Giovanni di Dio e Ruggi d’Aragona” University Hospital, Salerno, Italy; 5 Department of Electrical Engineering and Information Technology, University of Study of Naples “Federico II”, Naples, Italy; 6 Interdepartmental Center for Research in Healthcare Management and Innovation in Healthcare (CIRMIS), University of Naples “Federico II”, Naples, Italy

**Keywords:** Appendectomy, Multiple linear regression, Length of stay, Public health

## Abstract

**Background:**

The rapid growth in the complexity of services and stringent quality requirements present a challenge to all healthcare facilities, especially from an economic perspective. The goal is to implement different strategies that allows to enhance and obtain health processes closer to standards. The Length Of Stay (LOS) is a very useful parameter for the management of services within the hospital and is an index evaluated for the management of costs. In fact, a patient's LOS can be affected by a number of factors, including their particular condition, medical history, or medical needs. To reduce and better manage the LOS it is necessary to be able to predict this value.

**Methods:**

In this study, a predictive model was built for the total LOS of patients undergoing laparoscopic appendectomy, one of the most common emergency procedures. Demographic and clinical data of the 357 patients admitted at “San Giovanni di Dio e Ruggi d’Aragona” University Hospital of Salerno (Italy) had used as independent variable of the multiple linear regression model.

**Results:**

The obtained model had an R^2^ value of 0.570 and, among the independent variables, the significant variables that most influence the total LOS were Age, Pre-operative LOS, Presence of Complication and Complicated diagnosis.

**Conclusion:**

This work designed an effective and automated strategy for improving the prediction of LOS, that can be useful for enhancing the preoperative pathways. In this way it is possible to characterize the demand and to be able to estimate a priori the occupation of the beds and other related hospital resources.

## Introduction

The appendix is a protrusion of the large intestine, located where the large intestine joins the small intestine. The appendix performs some immunological functions, but it is not a fundamental organ [1]. When something, such as undigested food residues obstruct the internal lumen, it inflames, causing the "appendicitis".

In emergency surgery, one of the most common conditions that require a surgery is appendicitis [2]. Appendicitis is primarily a disease of adolescents and young adults with a peak incidence in the second and third decades of life. There is a slight male preponderance of 3:2 in teenagers and young adults. In adults, the incidence of appendicitis is approximately 1.4 times greater in men than in women [3]. In general, the risk for men and women is estimated at 8.6% and 6.7%, respectively [4]. Then, on 100,000 case of acute appendicitis, a range between 114.44 and 481.60 require a surgical procedure [5]. This value is a function of the socioeconomic level of the countries considered, in fact, the risk of appendicitis is rising sharply, especially in industrialized countries.

In the post-war period, thanks to the use of antibiotics and in particular penicillin, mortality was reduced (from over 40–2%). In the case of uncomplicated diagnosis, mortality is 0.08–0.4% while it rises to 12% in the case of perforation [6]. The diagnosis of acute appendicitis is predominantly clinical, in that is based on the accurate evaluation of the data provided by the anamnestic collection and on the patient's physical examination. It can be difficult, occasionally taxing the diagnostic skills of even the most experienced surgeon [7]. Early diagnosis is an essential condition for an effective treatment.

Appendectomy is a surgical procedure that can basically be performed in two ways: laparoscopic appendectomy (LA) and open appendectomy (OA). Both procedures can be decisive, and the choice is conditioned in the first place by the patient's age and the severity of appendicitis, also by the surgeon's skills and the availability of hospital resources [8].

Since its introduction in 1983, LA has quickly become a common and more adopted practice [9]. Nguyen et al. showed both an increased used of LA compared of OA and that patients undergoing LA have generally a no complicate diagnosis, a shorter length of stay (LOS) and fewer post-operative complications, without the increasing of healthcare costs [10]. Kwok KayYau et al., instead, showed the efficacy of LA in the complicated appendicitis [11]. LA proves once again to be feasible and safe, with a significantly shorter operative time, lower incidence of wound infection, and reduced LOS compared with OA.

The LOS—measured in days—is defined as the difference between the date of admission and the date of discharge of the patient. It is linked to the severity of the medical conditions, age of patient and any complication of the medical diagnosis, or the treatment received [12].

LOS is useful for planning admission and so a direct indicator of effectiveness and efficiency that has an impact on the organization and costs. For these reasons, in literature there are many works that have used LOS as an indicator of quality [13–15]. In all aspects of the healthcare sector, the extraction of clinical and organizational data for advanced analysis [16–19] and for process improvement [20–23] has proven to be a fundamental support in patient management.

LOS modeling is also not new in the literature. Verburg et al. [24] compared the performance of eight regression models when predicting intensive care unit LOS, failing to obtain optimal results for any of them, while Lee et al. [25] show the high performance of robust gamma mixed regression for the study of pediatric LOS. In addition to regression models, multiple linear regression was used to predict the LOS for patients undergoing valvuloplasty by considering their characteristics [26]. Austin et al. [27] use statistical analysis or analyzing LOS in a cohort of patients undergoing CABG surgery, while Scala et al. [28] show the benefits of implementing classifiers for predicting LOS [29–33].

In this study, a predictive model of the hospital stay of patients undergoing laparoscopic appendectomy was constructed to study how certain clinical and demographic variables affect the LOS prediction. The present research work is an extension of our previous work [34] in which the dataset considered was extended both in terms of years of observation and comorbidities considered, also evaluating the impact of comorbidities. The model used is multiple linear regression, which has proven effective in different healthcare implementations.

## Methods

The dataset, used in this study, included the information of 357 patients who have undergone an appendectomy in the five years 2016–2020 at the University Hospital “San Giovanni di Dio e Ruggi d’Aragona” of Salerno (Italy). The following variables was extracted from the hospital information system QuaniSDO:

- Gender (Male / Female);
- Age;
- Comorbidities;
- Diagnostic Related Group (DRG);
- Date of admission, discharge and LC procedure;

From these, the independent and dependent variables of the MLR model were obtained. In particular, from the analysis of DRG it was possible to identify if a patient had Complications during surgery or Complicated diagnosis. From the date, the pre-operative LOS (date of LC procedure—date of admission) and the total LOS was calculated. From the comorbidities, the following additional independent variables have been defined:

- Presence of comorbidities (yes / no);
- Heart Disease (yes / no);
- Diabetes (yes / no);
- Hypertension (yes / no);
- Obesity (yes / no);
- Peritonitis (yes / no);
- Cancer (yes / no).

Table 1 shows the distribution of the features into the sample.

**Table 1:** Features of dataset

Features	Dataset (N = 357)
*Gender*	
M	208 (58.3%)
F	149 (41.7%)
*Age*	
Age ≤ 40	246 (68.9%)
40 < Age ≤ 65	80 (22.4%)
Age > 65	31 (8.7%)
*Presence of comorbidities*	
Yes	82 (23%)
No	275 (77%)
*Complications during surgery*	
Yes	29 (8.1%)
No	328 (91.9%)
*Complicated diagnosis*	
Yes	146 (40.9%)
No	211 (59.1%)
*Pre-operative LOS*	
Mean	0.72
*LOS*	
Mean	4.83

The frequency of the groups of identified comorbidities on the population was calculated (Table 2). Frequency is a measure of the frequency of a disease or health condition in a population at a particular point in time [35], in this case in the five years 2016–2020.

**Table 2:** Frequency of comorbidities

Comorbidity	Frequency (%)
Heart disease	2.2
Diabetes	1.7
Hypertension	5.0
Obesity	1.4
Peritonitis	2.5
Cancer	0.6

IBM SPSS (Statistical Package for Social Science) ver. 27 was used to build a MLR model used to predict the total LOS [36].

### Multiple linear regression

In the last years, several data analytics methodologies have been proposed for supporting different applications [37, 38]. One of the most used one is the Multiple Linear Regression, that is a statistical technique that uses several explanatory variables to predict the outcome of a response variable. Multiple linear regression represents an extension of the simple linear regression model that uses just one explanatory variable. In this work, MLR model was implemented to predict the value of dependent variable Y (total LOS) starting from knowledge of several independent variables (Age, Gender, Pre-operative LOS, Complications during surgery, Complicated diagnosis, Presence of comorbidities, Heart Disease, Diabetes, Hypertension, Obesity, Peritonitis and Cancer).

The equation for a multiple linear regression is:

$$y = \beta_{0} + \beta_{1} x_{1} + \beta_{2} x_{2} + \beta_{3} x_{3} + \beta_{4} x_{4} + \ldots + \beta_{12} x_{12} + \varepsilon$$

where Y is the total LOS, β_0_ is intercept value, x_i_ are the twelve independent variables (pre-operative LOS, presence of complications, complicated diagnosis, gender, age, presence of comorbidities, heart disease, diabetes, hypertension, obesity, peritonitis and cancer) and β_i_ are the estimated regression coefficients of respective independent variables. $\varepsilon$ is the model error, i.e. the variation of our estimate of Y with respect to the real value. Before creating the model, the following six hypotheses must be verified:

1. The linear relationship between the independent and dependent variable. It can be checked through the scatter plot.
2. Absence of multicollinearity. Multicollinearity determines important changes in the values of the regression coefficients. Tolerance = 1-$R_{i}^{2}$ and Variance Inflation Factor (VIF) = $\frac{1}{{1 - R_{i}^{2} }}$—where $R_{i}^{2}$ is the proportion of the variation in the dependent variable that is predictable from the independent variables—are used to verify this assumption.
3. The independence of the residuals. In this case, the result of Durbin-Watson statistical test is analyzed.
4. The residuals have constant variance. It is possible to verify it by building the graph of "standardized residuals" against the "standardized predicted value".
5. The residuals are normally distributed. To verify this assumption a quantile–quantile (Q-Q) plot can be used.
6. Presence of outliers. The Cook's distance values always less than 1 guarantees the absence of outliers.

As a measure of the goodness of fit of a multiple regression model, the coefficient of determination, known as R^2^, is used. The linear determination index R^2^ represents the fraction of variance of Y which is explainable by the X regressors included in the model.

$$R^{2} = \frac{{Dev \left[ {\hat{Y}} \right]}}{Dev \left[ Y \right]}$$

R^2^ shows how well the terms (data points) fit a curve or line but there is also Adjusted-R^2^ that indicates how well terms fit a curve or line, but adjusts for the number of terms in a model. This is why in multiple linear regression with several predictors it is advisable to observe Adjusted-R^2^ [39].

$$Adjusted - R^{2} = 1 - \frac{n - 1}{{n - m - 1}} \left( {1 - R^{2} } \right)$$

where n represents the total sample size and m is the number of predictors. In most cases it turns out: 0 ≥ R^2^ ≥ 1. The $R^{2}$ and $\overline{{R^{2} }}$ tell whether the regressors are suitable for predicting the values of the dependent variable in the sample of data used. If $R^{2}$ (or $\overline{{R^{2} }}$) tends to one, the regressors produce good predictions of the dependent variable, if $R^{2}$ (or $\overline{{R^{2} }}$) tends to 0 the opposite is true. The level of significant α is equal to 0.05.

## Results

Before building the MLR model, the six hypotheses were tested. The result of Durbin-Watson test was 1.505 and it was between the acceptable range of [1.5; 2.5] to demonstrate the independence of residual. The Cook’s distance for each observation was less than 1, so there were not outliers in the dataset that negatively affect the estimate of the coefficients. For the 2nd assumption, Table 3 shows the values of VIF, and Tolerance obtained for each independent variable.

**Table 3:** Collinearity statistics

Input variable	Tolerance	VIF
Pre-operative LOS	0.921	1.086
Presence of complications	0.484	2.066
Complicated diagnosis	0.869	1.151
Gender	0.895	1.117
Age	0.632	1.583
Presence of comorbidities	0.543	1.842
Heart disease	0.693	1.444
Diabetes	0.736	1.358
Hypertension	0.748	1.337
Obesity	0.915	1.093
Peritonitis	0.639	1.565
Cancer	0.943	1.060

The VIF values were always less than 10 and the Tolerance values were always greater than 0.2, so the absence of multicollinearity was verified.

**Fig. 1:**
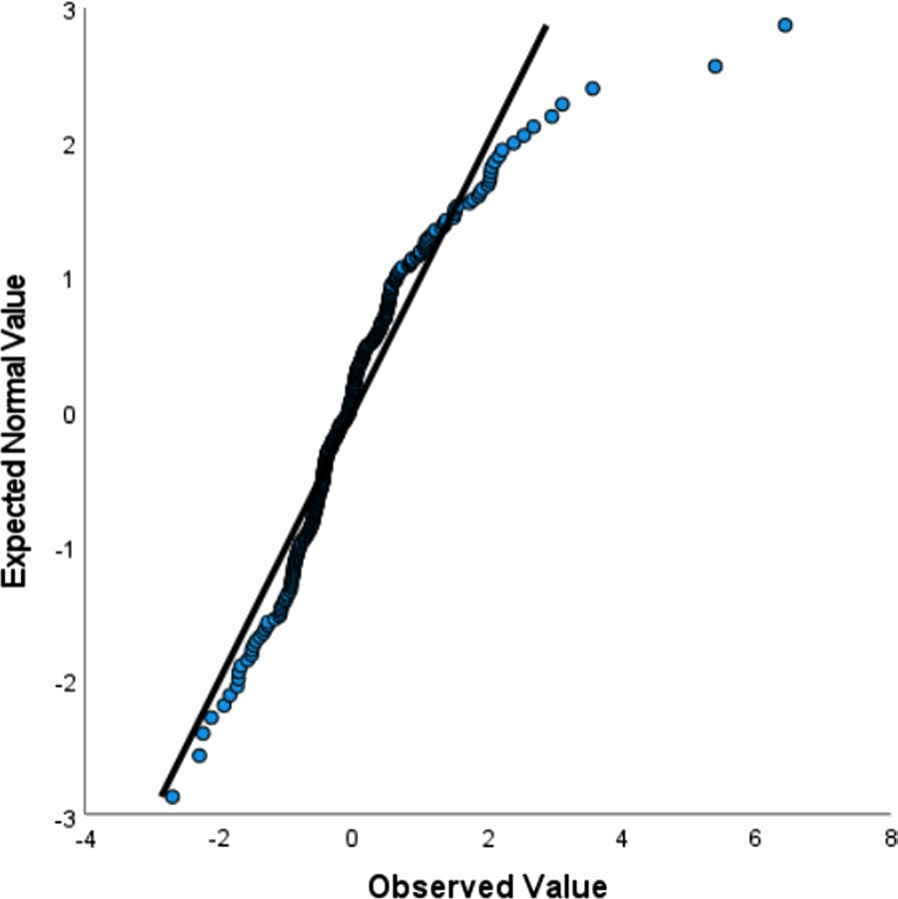
Normal Q-Q Plot of Standardized Residual

Figure 1 shows the Q-Q plot, a graph “observed value” against “expected normal value” used to test the normally distribution of the residual values.

As can be seen from the Fig. 1, the points are quite close to the line. There are few outliers, but which is proven not to affect the goodness of the coefficients estimation. In fact, Cook's distance was calculated for each point and the maximum value obtained was 0.8, which is well below the required threshold 1.

**Fig. 2:**
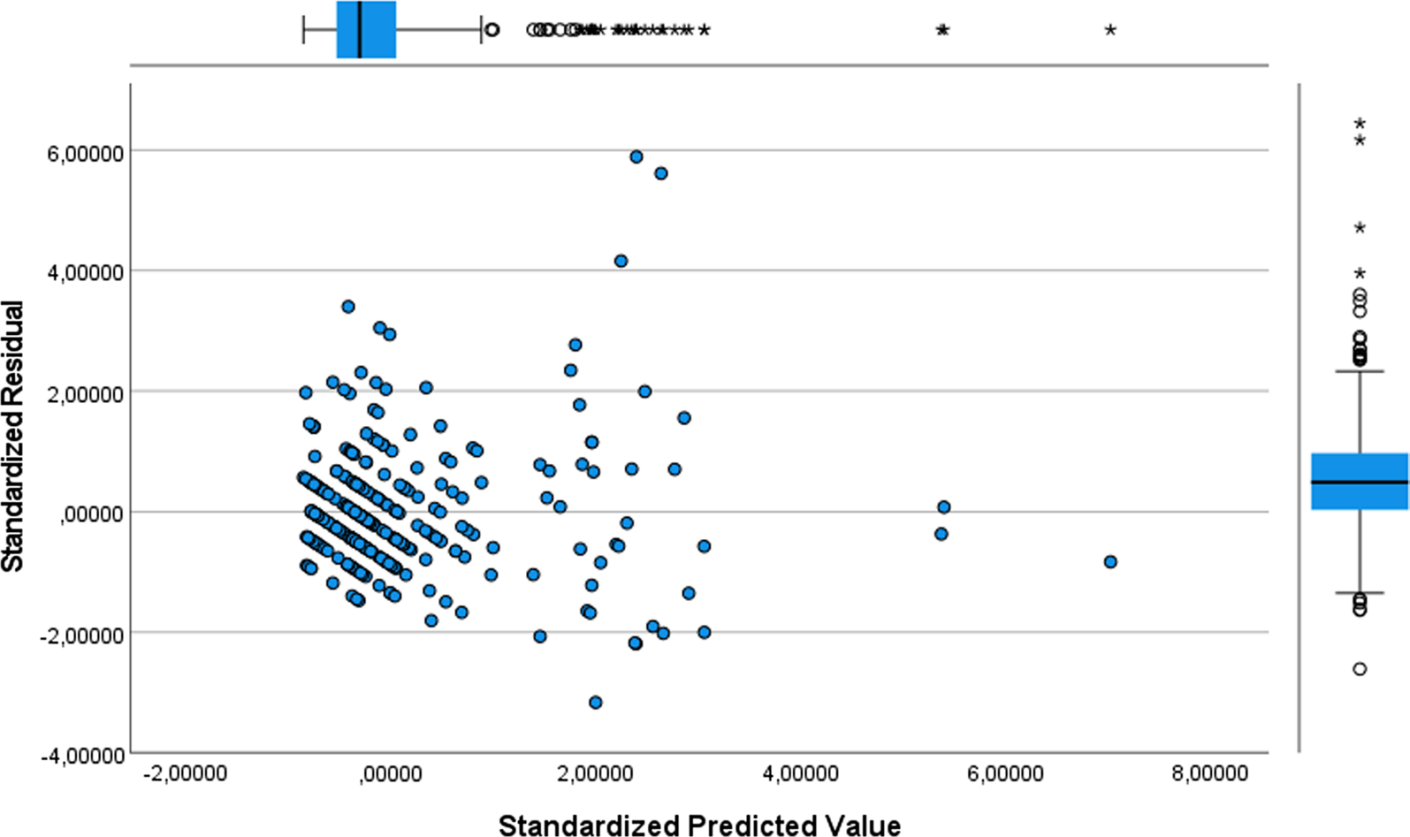
Plot of "standardized residuals" against the "standardized predicted value"

Figure 2 shows the graph of "standardized residuals" against the "standardized predicted value" used to verify that the variance of the residuals is constant.

The variance of residuals was not constant across predicted values, so there was a moderate violation of homoscedasticity, which was however considered acceptable. In fact, Table 4 shows that the analysis of variance is significant, i.e. there is indeed a linear dependence between the dependent variable and the regressor variable (p-value < 0.05). Then, the MLR model was implemented. Table 4 shows the performance of the model.

**Table 4:** Model summary and Fisher's exact test

Model	R	R^2^	Adjusted—R^2^	Std. Error of the Estimate	Sum of squares	Degrees of freedom	Mean square	F	p-value
Regression	0.764	0.584	0.570	2.026	1984.572	12	165.381	40.272	** < 0.0001**
Residue					1412.678	344	4.107	-	
Tot					3397.249	356		-	

The coefficient of determination (R^2^) was greater than 0.5 so it can be considered a good preliminary model to represent the problem. The p-values below the alpha value are highlighted in bold.

Table 5 shows the coefficients of the model and the results of the t-test, used to study the significance of the regression coefficients (βi). P-values < 0.05 were considered statistically significant.

**Table 5:** Standardized and Unstandardized coefficients with p-values of the MLR analysis

Variable	Unstandardized coefficients		Standardized coefficients beta	t	p-value
	B	Std. error			
Intercept	7.542	0.760	–	9.919	**0.000**
Pre-operative LOS	0.941	0.066	0.516	14.240	**0.000**
Presence of complications	− 3.949	0.573	− 0.344	− 6.887	**0.000**
Complicated diagnosis	− 0.863	0.234	− 0.137	− 3.684	**0.000**
Gender	− 0.160	0.230	− 0.026	− 0.696	0.487
Age	0.024	0.007	0.148	3.393	**0.001**
Presence of comorbidities	0.740	0.346	0.101	2.139	0.033
Heart disease	0.237	0.871	0.011	0.272	0.786
Diabetes	− 1.861	0.972	− 0.078	− 1.913	0.057
Hypertension	1.053	0.563	0.075	1.857	0.064
Obesity	− 0.911	0.954	− 0.035	− 0.954	0.341
Peritonitis	− 0.649	0.856	− 0.033	− 0.758	0.449
Cancer	− 1.998	1.480	− 0.048	− 1.350	0.178

The p-value was less than 0.05 for the Pre-operative LOS, the Presence of complication, Complicated diagnosis and Age. Among these variables that significantly influence LOS, the pre-operative LOS has the highest coefficient in accordance with the definition of total LOS (pre-operative LOS + post-operative LOS).

## Discussion

The aim of this work was to build a predictive model, using the multiple linear regression, of the total LOS for patients undergoing a laparoscopic appendectomy at "San Giovanni di Dio e Ruggi d’Aragona" University Hospital of Salerno (Italy) in the five-year period 2016–2020. Starting from a group of selected information (Gender, Age, Comorbidities, Diagnostic Related Group (DRG), Date of admission, Date of discharge and Date of LC procedure) the independent variables of the model were obtained. In particular, the analysis of the comorbidities made it possible to divide patients into subgroups by categories of pathologies with higher frequency in our sample.

A simple model has been obtained with a value of R^2^ equal to 0.570. The value of R^2^, even if slightly, exceeds the value of 0.5 that support its use for this task. In fact, the linear models have the advantage of being easy to understand and use during the activities carried out by healthcare staff. The results of t-test demonstrate that Pre-operative LOS, Presence of complication, Complicated Diagnosis and Age are the variables that most influence the total LOS. The Pre-operative LOS is a value that we expected because it is linked with the definition of LOS. The result of the influences is actually in line with what can be read from the literature on the topic. For example, Liu et al. [40] show how age is a factor influencing procedures related to 18 different DRGs. Remaining in the theme of appendectomy, Ponsiglione et al. [41] showed how in procedures performed in urgency there is a strong link between LOS and comorbidities, while Demir et al. [42] highlight how both postoperative and total LOS of the patients undergoing appendectomy are more likely to be affected by patients' demographic characteristics and clinical needs. In addition, other variables not included in this study have significant effects on LOS. For example, Crandall et al. [43] showed as the operative time of day was a surprisingly important determinant of hospital LOS while Cheong et al. [44] highlighted a significantly longer hospital stay was associated with open appendectomy, pediatric surgeon, and the Territories for simple appendicitis in pediatric patients.

The multi-year study showed a dependence of total LOS on age that was not evident in the previous model [30]. This information is important for the possible creation of pathways for specific age groups, for the management of complications or for the standardization of the pre-operative phase, as already done by the hospital for femur fracture in patients older than 65 years [45].

This work demonstrated that the MLR represents a valid preliminary support to characterize the demand and to be able to estimate a priori the occupation of the beds and the use of other hospital resources.

Although the work is novel in terms of sample size and number of comorbidities analyzed, it is not without limitations. In particular, the model is not validated through the use of datasets from other hospitals, the impact that other procedures, such as those related to possible complications, may have on LOS is not included, and the value of R.^2^ is slightly above the 0.5 value and this makes it necessary to search for a more robust predictive model. For example, classification algorithms (such as Logistic Regression) could be a valid alternative [46].

## Conclusion

In this work, the data of 357 patients undergoing LC at "San Giovanni di Dio e Ruggi d’Aragona" University Hospital of Salerno (Italy) in the five-year period 2016–2020 was study using MLR model, whose aim is to predict LOS on the basis of patients' clinical and demographic variables. Among the independent variables, Pre-operative LOS, presence of complication, complicated diagnosis and age are the variables that most influence the total LOS. The results are in line with what can be found in the scientific literature, in which the impact of age, complicated diagnoses, and complications is discussed for several clinical procedures including appendectomy. The model, in addition, has good performance that validates it as a prediction tool to be given for use by clinicians. The linear model, however, although very simple in its interpretation, could not be robust enough. Therefore, future developments will include validation of the model with multicenter studies as well as the use of advanced data processing tools.

## Data Availability

The datasets generated and/or analyzed during the current study are not publicly available for privacy reasons but are available from the corresponding author on reasonable request.

## Abbreviations

LC: Laparoscopic Appendectomy
OA: Open Appendectomy
LOS: Length Of Stay
